# 

**Published:** 1898-07-30

**Authors:** 


**Notices of Births, Deaths, and Marriages** are inserted in this column at a charge of 2s. 6d. for an announcement not exceeding four lines.

### NOTICE TO CORRESPONDENTS.

All MS., Letters, Books for Review, and other matters intended for the Editor should be addressed The Editor, "The Hospital," The Hospital Building, 28 & 29, Southampton Street, Strand, W.C.

The Editor cannot undertake to return rejected MS., even when accompanied by stamped directed envelope.

**Notice to Advertisers and Subscribers.**

All Advertisements, Orders for Copies of The Hospital, and Business Communications should be addressed to **THE MANAGER, (Not to The Editor),** The Hospital, The Hospital Building, 28 & 29, Southampton Street, Strand, London, W.C.

The Cost of Subscription, post free, is as follows:—Per week, 2½d. per quarter, 2s. 8d.; per half-year, 5s. 3d.; per annum, 10s. 6d. Foreign:—Per annum, 15s. 2d.; payable, in all cases, in advance.

**NOTICE.—Vol. XXIII. of "The Hospital" (October, 1897, to March, 1898), in handsome cloth binding, is now on sale at the Office of this Journal, price 7s. 6d. Cases for binding the Numbers contained in this Volume, 1s. 6d. Index to Vol. XXIII., 2½d., post free.**

**The Monthly Part for August will be ready on August 2nd. Price 6d., by post 8d.**

### BOOKS RECEIVED.

SIMPKIN, MARSHALL, AND CO.

"Billy," and Other Stories. By Henry Clarke.

REBMAN PUBLISHING COMPANY.

"An Atlas of Clinical Diagnosis." By Dr. Christfried Jakob. Edited by Augustus A. Eshner, M.D.

CHATTO AND WINDUS.

"Foods for the Fat." By N. E. Yorke Davies.

### THE STUDENT OF MEDICINE.

### APPOINTMENTS.

J. B. Cooke, L.R.C.P., L.R.C.S.Edin., appointed Medical Officer to H.M. Convict Prison, Borstal, Kent. Dr. G. C. Cory, appointed Government Medical Officer at Bingara, New South Wales. W. Corry, M.D., appointed Health Officer for the Shire of Phillip Island and Woolamai, Victoria. H. O. Cowen, M.B., appointed Health Officer for the Borough of Reywood, Victoria. E. O. Cox, L.R.C.P., L.R.C.S.Edin., L.F.P.S.Glasg., appointed Medical Officer for the Second District of the Cleobury Mortimer Union. A. E. Ffrost, M.B., B.S., appointed Clinical Assistant to the Chelsea Hospital for Women. H. C. Goodman, M.B., B.C.Camb., M.R.C.S.Eng., L.R.C.P.Lond., appointed Resident Medical Officer to Kasr-el-Aini Hospital. Dr. P. J. Hamilton, appointed Medical Officer to the Ardara Dispensary District, co. Donegal. L. de C. Harston, L.R.C.P.Edin., M.R.C.S.Eng., appointed Medical Officer for the Workhouse and No. 8 and 9 Districts of the Kingsbridge Union. G. S. Hatton, M.D., M.S Durh., F.R.C.S.E., appointed an Honorary Surgeon to the North Staffordshire Infirmary, Hartshill, Stoke-on-Trent. C. A. Hill, M.B., B.C., B.A.Cantab., M.R.C.S.Eng., L.R.C.P.Lond., appointed Stipendiary Medical Officer to the Liverpool Hospital for Consumption and Diseases of the Chest. F. A. S. Hutchinson, M.B., B.C.Camb., M.R.C.S.Eng., L.R.C.P.Lond., appointed Resident Surgical Officer to the Kasr el-Aini Hospital. E. Jeffery, L.R.C.P., L.R.C.S.Edin., appointed Medical Officer for the Second District of the St. Austell Union. H. C. Lawrence, L.R.C.P.Lond., &c., appointed Honorary Physician to the Home for Sick Children, Cheltenham. T. C. Lawson, M.R.C.S., appointed Medical Officer for the Stogumber District of the Williton Union. F. C. Madden, M.B., B.S.Melb., F.R.C.S.Eng., Medical Superintendent of the Hospital for Sick Children, Great Ormond Street, appointed Professor of Surgery at the Egyptian Government School of Medicine, and Senior Surgeon to the Kasr-el-Aini Hospital, Cairo. T. Massie, M.B., appointed by the School Board for London Lecturer on Ambulance at the Evening Continuation Schools in Southwark, Walworth, Newington, and Bermondsey. F. H. May, L.R.C.P.Lond., D.P.H. (Fac.P. & S.Glasg.), appointed Medical Officer of Health to the Aston Manor Urban District Council. F. R. S. Milton, M.R.C.S.-Eng., L.R.C.P.Lond., appointed Professor of Clinical Surgery at the School, and Surgeon to the Kasr-el-Aini Hospital. H. P. Noble, M.B., B.S.Lond., M.R.C.S., L.R.C.P., appointed Honorary Anæsthetist to Children's Hospital, Paddington Green. G. Palmer, M.B., appointed Health Officer for the Shire of Ararat, Victoria. J. C. Potter, M.B., C.M.Edin., appointed Medical Officer for the Tudhoe District of the Durham Union. R. U. Russell, L.R.C.P.Edin., appointed Medical Superintendent for Insane, and Health Officer for the Port of Newcastle, New South Wales. S. Riseley, M.D., C.M.Edin., appointed Honorary Ophthalmic Surgeon to the Sheffield Royal Hospital.

